# A Global Survey on General Surgery Resident Involvement in Metabolic and Bariatric Surgery

**DOI:** 10.7759/cureus.91329

**Published:** 2025-08-31

**Authors:** Adam Abu-Abeid, Wah Yang, Jonathan B Yuval

**Affiliations:** 1 Division of General Surgery, Tel Aviv Sourasky Medical Center, Tel Aviv, ISR; 2 Faculty of Medicine, Tel Aviv University, Tel Aviv, ISR; 3 Department of Surgery, The First Affiliated Hospital of Jinan University, Guangzhou, CHN

**Keywords:** bariatric surgery, international survey, metabolic surgery, residents, training

## Abstract

Background: There is limited global information on the involvement and autonomy of general surgery residents in metabolic and bariatric surgery (MBS).

Methods: We conducted an international online survey from July 15 to September 15, 2024, assessing residents’ participation in MBS procedures, outpatient clinics, simulation courses, mentorship, and research. The 40-question survey was distributed via social media, email lists, and the Global Obesity Collaborative Group.

Results: A total of 198 general surgery residents from all continents participated, primarily from Europe (51%), Asia-Pacific (22%), and Latin America (13%). The mean age was 34.0 ± 6.7 years, and 31% were women. The average postgraduate year was 4.11 ± 3.22 years. Most respondents (68%) intended to pursue an MBS fellowship. One-third lacked MBS services at their institution. However, 83% reported acting as primary surgeons, primarily in sleeve gastrectomy (47%), Roux-en-Y gastric bypass (RYGB) (25%), and one anastomosis gastric bypass (OAGB) (22%). Additionally, 71% participated in outpatient MBS-related clinics. Mentorship was common (81%), with 49% mentored by an MBS surgeon. Research engagement was high, with 85% publishing at least one article, 45% in MBS, and 30% presenting at international conferences. MBS-dedicated services and high-volume centers were more frequent in North and Latin America and less common in other regions.

Conclusions: This is the largest international survey on general surgery residents’ involvement in MBS. These findings provide insight into global training practices and highlight the need for improved exposure to MBS worldwide.

## Introduction

Metabolic and bariatric surgery (MBS) is the most effective treatment for obesity and its associated comorbidities [1]. Given its well-established benefits [2-4], the global volume of MBS has risen steadily over recent decades [5,6]. However, the adoption of specific procedures varies widely across the five chapters of the International Federation for the Surgery of Obesity and Metabolic Disorders (IFSO) [6].

This growing prevalence of MBS has altered the landscape of surgical training, shaping the patient population and procedures encountered by general surgery residents. As more patients undergo MBS, it is increasingly important that all physicians, particularly fully trained surgeons who do not specialize in MBS, are equipped to recognize the unique challenges, complications, and presentations of these patients in clinical practice. Adequate exposure to, and participation in, MBS procedures and perioperative care during residency are essential for developing the skills needed to manage this complex patient population [7].

The aim of this study was to evaluate global patterns of general surgery residents’ exposure to MBS, including operative experience, outpatient clinic participation, and academic or research involvement. We conducted an online survey distributed through social media, email lists, and the Global Obesity Collaborative Group. We also compared training experiences across global regions to identify differences in exposure to key aspects of MBS care. Such insights could inform future international collaboration and exchange programs aimed at improving MBS training worldwide.

## Materials and methods

An online survey was open for completion between July 15, 2024, and September 15, 2024, and included 40 open and closed-ended questions regarding respondents' demographics, workplace, exposure, and participation in MBS procedures, involvement in outpatient MBS care, and participation in research in general and the field of MBS (Appendix A). The survey was written by AAA and edited by JBY and WY. To complete the survey, respondents were required to provide their name and a functioning email address. The complete questionnaire can be seen in the supplementary materials. The survey was disseminated online on the websites and email lists of the Global Obesity Collaborative and in social media groups related to general and bariatric surgery. The nature of the study does not mandate institutional review board or informed consent of participants at the medical centers and universities of the authors, and was explicitly waived by the institutional review boards. Completion of the questionnaire was considered as implied consent of the respondents. 

Each participant was assigned or asked to provide a unique email address that, when combined with the demographic data provided, created a unique fingerprint. This allowed us to track individual participation.

The survey instrument did not undergo any pilot testing or validation prior to dissemination. Regular reminders were posted on the platforms and channels where the study was initially disseminated. These reminders reiterated the importance of participation and the study's closing date.

Inclusion and exclusion criteria were as follows: respondents were included in the study if they were medical doctors or of equivalent degree (e.g., doctors of osteopathy) enrolled in a general surgery residency. Attending general surgeons, residents in other (non-general surgery) residency programs, and doctors not yet enrolled in a residency program were excluded.

High-volume centers were considered as centers performing 100 or more MBS procedures yearly. 

Categorical variables are presented as numbers and percentages, and continuous variables as mean ± standard deviation. Some information is also displayed graphically and/or descriptively. For comparison between groups, the chi-square (χ2) or Student's t-test was used. To compare the six different geographical regions, χ² and ANOVA were used as appropriate. P-values < 0.05 were considered statistically significant. The data were assessed for the assumptions of the chosen tests. Normality of the data distributions was evaluated using both the Shapiro-Wilk test and visual inspection of Q-Q plots. For data where the assumption of normality was met, we proceeded with Student's t-tests for two-group comparisons and ANOVA for three or more groups. For data where the normality assumption was violated, appropriate non-parametric alternatives were utilized, specifically the Mann-Whitney U test for two-group comparisons and the Kruskal-Wallis test for three or more groups. Corrections for multiple assumptions were not carried out. Analyses were performed using IBM SPSS Statistics software, version 29 (IBM Corp., Armonk, NY, USA).

## Results

**Figure 1:**
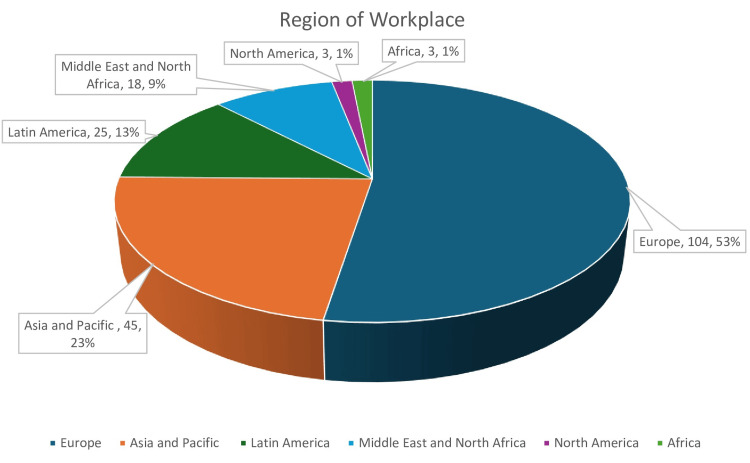
Distribution of resident survey respondents

A total of 223 physicians completed the survey. Of these, 25 were excluded, 24 were attending general surgeons, and one was excluded because they were not an intern or resident. The study cohort was made up of 198 general surgery residents from 35 countries (Figure 1). Because of the open nature of the dissemination of the questionnaire, the number of residents exposed to the survey who did not respond is unknown.

Responding residents' demographics can be seen in Table 1. Respondents hailed from all five continents. Most commonly, residents were from the following regions, in descending order: Europe (51%), Asia and the Pacific (22%), Latin America (13%), the Middle East and North Africa (10%), North America (2%), sub-Saharan Africa (1%), and other regions (1%). The mean age of respondents was 34.0 ± 6.71 years, and 31% were women. The mean duration of general surgery residency was 5.11 ± 1.53 years, and the mean postgraduate year of responding residents was 4.11 ± 3.22 years. Among the study cohort, residents were most interested in the following subspecialties, in decreasing order: MBS (52%), upper gastrointestinal (GI) tract surgery (40%), surgical oncology (33%), colorectal surgery (31%), abdominal wall and hernia surgery (30%), hepatic-pancreatic-biliary surgery (25%), breast surgery (13%), endocrine surgery (10%), and other subspecialties (11%). More than two-thirds of the study cohort (68%) considered completing an MBS fellowship.

**Table 1:** Demographic Characteristics of the General Surgery Residents MBS: metabolic and bariatric surgery; UGI: upper gastrointestinal; CRS: colorectal surgery; SO: surgical oncology; AWS: abdominal wall surgery; HPB: hepato-pancreatico-biliary *-Participants could select more than one response; +-N =180 relevant responses

Parameter	Number (%) or Mean ± Standard Deviation
Female Sex	61 (30.8%)
Age (years)	34.0 ± 6.71
Region of Workplace:	
Europe	104 (52.5%)
Asia and Pacific	45 (22.7%)
Latin America	25 (12.6%)
Middle East and North Africa	18 (9.09%)
North America	3 (1.52%)
Africa	3 (1.52%)
Length of General Surgery Residency (years)	5.11 ± 1.53
Current Postgraduate Year^+^	4.11 ± 3.22
Most Interested in the Following Subspecialty*:	
MBS	102 (51.5%)
UGI	79 (39.9%)
SO	65 (32.8%)
CRS	62 (31.3%)
AWS	59 (29.8%)
HPB	50 (25.3%)
Breast	26 (13.1%)
Endocrine	19 (9.60%)
Other	21 (10.6%)
Considering Completing an MBS Fellowship	134 (67.7%)

Information regarding residents' workplaces and participation in MBS procedures and care can be seen in Table 2. More than a quarter of residents (28%) had no MBS service at their hospital. More than half of the respondents (59%) were trained in low-volume centers performing 100 or fewer MBS procedures a year. However, over three-quarters (77%) of residents had participated in MBS procedures at their workplace. The vast majority of respondents (83%) had participated as primary surgeons in MBS procedures (either leading the surgery or performing the majority of the case), most commonly, and in decreasing order, sleeve gastrectomy (SG): 47%, Roux-en-Y gastric bypass (RYGB): 25%, one anastomosis gastric bypass (OAGB): 22%, single anastomosis duodeno-ileal bypass (6%), biliopancreatic diversion (3%), gastric banding (3%), and duodenal switch (1%). Around one-fifth of respondents (21%) had participated in more than 100 MBS procedures. Detailed performance/completion of specific steps of various MBS procedures by respondents can be seen in the supplementary materials (Appendix B-D). Most respondents had participated in MBS outpatient care (71% preoperative, 71% postoperative), and almost all (92%) believed that participation in MBS should be mandatory during surgical residency. Most residents had a surgical mentor (81%), and the mentor was an MBS surgeon for nearly half (49%).

**Table 2:** Workplace and Participation in MBS MBS: metabolic and bariatric surgery

Parameter	Number (%)
Presence of MBS Service at the Workplace	143 (72.2%)
MBS Procedures Performed Annually at the Workplace	
0	30 (15.2%)
1-50	45 (22.7%)
51-100	41 (20.7%)
101-200	34 (17.2%)
201-500	30 (15.2%)
501-1000	10 (5.05%)
>1000	8 (4.04%)
Have Participated in MBS at the Workplace	152 (76.8%)
MBS Procedure Experience	
0	26 (13.1%)
1-10	48 (24.2%)
11-30	28 (14.1%)
31-50	30 (15.2%)
51-100	25 (12.6%)
>100	41 (20.7%)
Proportion of MBS Procedures in Relation to All Surgeries Participated	
0%	25 (12.6%)
1%-10%	110 (55.6%)
11%-30%	32 (16.2%)
31%-50%	13 (6.57%)
>50%	18 (9.09%)
Participation as Primary Surgeon in MBS	165 (83.3%)
Participation as Primary Surgeon in	
Sleeve Gastrectomy	92 (46.5%)
Roux-en-Y Gastric Bypass	50 (25.3%)
One Anastomosis Gastric Bypass	43 (21.7%)
Single Anastomosis Duodeno-ileal Bypass	11 (5.56%)
Biliopancreatic Diversion	5 (2.53%)
Gastric Banding	5 (2.53%)
Duodenal Switch	1 (0.05%)
Completed any MBS-Related Surgical Training:	100 (50.5%)
Completed Cadaver Course	17 (8.59%)
Laparoscopic Simulator	64 (32.3%)
Laparoscopic Training Box	59 (29.8%)
Believe Participation in MBS is Mandatory in General Surgery Residency	182 (91.9%)
Have a Surgical Mentor	160 (80.8%)
Mentor is the MBS Surgeon	96 (48.5%)
Preoperative Clinic Participation	140 (70.7%)
Postoperative Clinic Participation	141 (71.2%)

Resident participation in research can be seen in Table 3. Nearly two-thirds of responding residents (65%) had a scientific mentor, and for nearly one-third (32%), the mentor was an MBS surgeon. The vast majority of respondents had published at least one publication (86%), almost one-half (45%) had published MBS-related research, and one-quarter (25%) of respondents had published at least 20 publications. Many respondents had previously presented at national (70%) and international (56%) scientific conferences, and a significant minority of respondents had previously presented MBS-related research at national (38%) and international (30%) conferences. 

**Table 3:** General Surgery Residents’ Academic Involvement MBS: metabolic and bariatric surgery

Parameter	Number (%)
Have a Scientific Mentor	129 (65.2%)
Mentor is MBS Surgeon	64 (32.3%)
Number of Publications	
0	29 (14.6%)
1-5	73 (36.9%)
6-10	31 (15.7%)
11-20	15 (7.58%)
>20	50 (25.3%)
Proportion of MBS-Related Publications	
0%	110 (55.6%)
1%-10%	37 (18.7%)
11%-30%	16 (8.08%)
31%-50%	9 (4.55%)
>50%	26 (13.1%)
Presentation at the National Meeting	138 (69.7%)
MBS Presentation at the National Meeting	76 (38.4%)
Presentation at the International Meeting	110 (55.6%)
MBS Presentation at the International Meeting	59 (29.8%)

**Figure 2:**
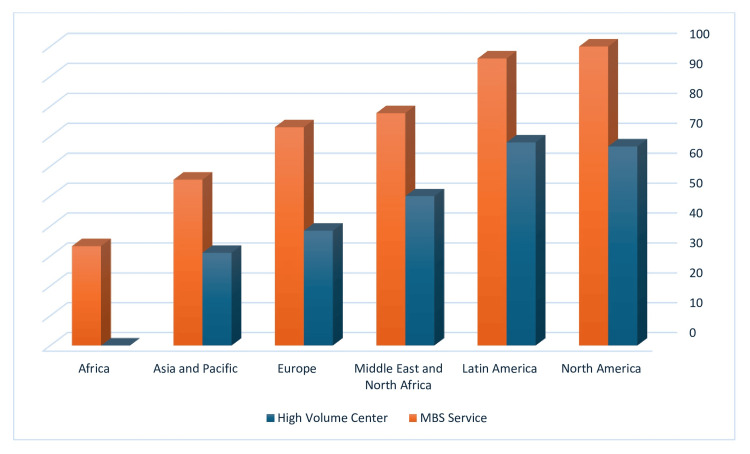
Percentage of high-volume centers and centers with a metabolic and bariatric surgery service (MBS) according to respondents from each region

A comparison of responses from different geographical areas can be seen in Table 4. The percentage of high-volume centers and centers with an MBS service in each region, according to residents' responses, can be seen in Figure 2. Meaningful differences were found in the length of general surgery residency between regions, which was shortest in Latin America (3.88±0.61 years) and longest in Europe (5.78±1.03 years). Meaningful differences were also found in the frequency of high-volume centers and MBS services between regions. Both were most frequent in North America (66.7%, 100%) and Latin America (68.0%, 96.0%), followed by (in decreasing order) the Middle East and North Africa (50%, 77.8%), Europe (38.5%, 73.1%), Asia and the Pacific (31.1%, 55.6%), and sub-Saharan Africa (0%, 33.3%, p=0.023 and 0.004, respectively). Groups were too small for additional meaningful subgroup breakdowns of significant results across multiple geographical groups.

**Table 4:** Comparison of MBS Involvement in Different Geographic Areas MBS: metabolic and bariatric surgery; SG: sleeve gastrectomy; RYGB: Roux-en-Y gastric bypass; OAGB: one anastomosis gastric bypass; *: ANOVA; **: χ2

Parameter N (%) or Mean ± Standard Deviation	Europe (N=104)	Asia and Pacific (N=45)	Latin America (N=25)	Middle East and North Africa (N=18)	North America (N= 3)	Africa (N=3)	p-value	Test Value
Female	38 (36.5)	9 (20.0)	7(28.0)	7(38.9)	0 (0.0)	0 (0.0)	0.194	7.38*
Age (years)	33.8±5.33	34.4±6.75	32.4±11.1	30.4±6.46	34.0±8.00	34.3±0.58	0.357	1.11**
Length of Residency (years)	5.78±1.03	4.39±1.58	3.88±0.61	5.22±2.86	5.00±0.00	5.00±1.00	<0.001	10.8**
PGY	4.58±2.59	4.03±4.91	2.41±1.22	3.88±3.77	3.33±2.08	4.00±1.00	0.132	1.72**
MBS Service at Workplace	76 (73.1)	25 (55.6)	24 (96.0)	14 (77.8)	3 (100)	1 (33.3)	0.004	17.0*
High Volume MBS Service	40 (38.5)	14 (31.1)	17 (68.0)	9 (50.0)	2 (66.7)	0 (0.0)	0.023	13.1*
Primary Surgeon SG	52 (50.0)	22 (48.9)	9 (36.0)	7 (38.9)	1 (33.3)	1 (33.3)	0.767	2.56*
Primary Surgeon RYGB	25 (24.0)	12 (26.7)	7 (28.0)	4 (22.2)	0 (0.0)	2 (66.7)	0.541	4.06*
Primary Surgeon OAGB	21 (20.2)	12 (26.7)	5 (20.0)	3 (16.7)	0 (0.0)	2 (66.7)	0.358	5.50*
Surgical Mentor	76 (73.1)	39 (86.7)	23 (92.0)	17 (94.4)	2 (66.7)	3 (100)	0.068	10.3*
MBS Surgical Mentor	41 (39.4)	25 (55.6)	19 (76.0)	9 (50.0)	1 (33.3)	1 (33.3)	0.136	14.9*
Scientific Mentor	66 (63.5)	27 (60.0)	17 (68.0)	15 (83.3)	2 (66.7)	2 (66.7)	0.643	3.37*
MBS Scientific Mentor	29 (27.9)	16 (35.6)	12 (48.0)	6 (33.3)	1 (33.3)	0 (0.0)	0.369	5.40*
Clinic Participation	80 (76.9)	35 (77.8)	17 (68)	17 (94.4)	3 (100)	3 (100)	0.296	6.11*
MBS Publications	42 (40.4)	21 (46.7)	14 (56.0)	7 (38.9)	2 (66.7)	1 (33.3)	0.685	3.01*
MBS Presentation International Meeting	27 (26.0)	19 (42.2)	9 (36.0)	3 (16.7)	1 (33.3)	0 (0.0)	0.200	7.29*

## Discussion

General surgery residency is among the most demanding training programs in medicine [8]. Over approximately five years [9], residents acquire a broad foundation of surgical knowledge and technical skills under the supervision of attending surgeons, gradually gaining independence and responsibility. Alongside clinical training, residents are expected to engage in academic activities, such as participating in clinical research and attending conferences. While residency demands significant time, mental focus, and emotional resilience, it ultimately prepares physicians to become competent, compassionate, and confident surgeons capable of managing complex cases in high-pressure settings.

The degree of resident involvement in MBS during training remains undefined. There is general consensus on the need to balance educational opportunities for residents with patient safety. Evidence suggests, however, that resident participation in MBS is safe and does not adversely affect postoperative outcomes [10].

This survey was conducted to assess the extent of general surgery residents’ exposure to MBS worldwide. Most respondents were from Europe and the Asia-Pacific region (73%) and expressed interest in pursuing MBS as a subspecialty or fellowship. Data from a multi-institutional study of 18 Accreditation Council for Graduate Medical Education (ACGME)-accredited general surgery residency programs in the United States showed that graduates performed more procedures in the field of their eventual fellowship during residency, a trend observed across all 12 fellowship specialties [11]. A similar retrospective analysis of ACGME case logs from 101 graduates at a single institution confirmed this pattern [12], emphasizing that subspecialization often begins during residency.

Concerns have been raised about whether resident involvement in MBS could increase complication rates. Valente et al. compared outcomes of 313 SG cases performed by senior MBS surgeons versus residents, finding no differences in postoperative complications, though operative times were longer for residents (74.3 vs 65.3 minutes) [13]. Similarly, Major et al. found no difference in postoperative outcomes for SG and RYGB cases, aside from longer operative times for resident-performed procedures [14]. In a patient-perception study, most patients supported residents observing surgery, but only 56% favored active participation [15]. Nevertheless, available evidence indicates that resident participation is safe and offers essential operative experience.

Previous surveys have focused primarily on early-career attending surgeons. Felsenreich et al. reported findings from the Young International Federation for the Surgery of Obesity and Metabolic Disorders (IFSO) survey, which included mostly attending surgeons (<45 years old), with SG being the most common procedure performed as the primary surgeon [7]. Common training modalities reported included laparoscopic box trainers (71%), dry labs (30%), animal courses (23%), and wet labs (21%). In our survey of residents, SG was also the most common primary surgeon procedure (46.5%), likely reflecting its global predominance. Over half (51%) had completed some form of MBS training, most often laparoscopic simulation (32%), laparoscopic box training (30%), or cadaver-based wet labs (9%), underscoring the potential of structured training to enhance technical proficiency.

Mentorship and academic involvement were also prevalent. In the Young IFSO survey, 85% of respondents participated in research, and 82% had a surgical mentor. Similarly, 81% of residents in our study had a surgical mentor, and 65% had a scientific mentor. Nearly half (49%) reported that their surgical mentor specialized in MBS, and 32% had a scientific mentor in MBS. Eighty-five percent had authored scientific publications, with 45% publishing on MBS-related topics. Mentorship plays a critical role in guiding residents toward academic success and fostering professional development [16].

Overall, our findings highlight both similarities and disparities in MBS training among general surgery residents worldwide. National and international collaborations, including clinical exchanges between regions with varying MBS volumes and procedural profiles, could help standardize and strengthen training. Such initiatives may provide residents from low-volume centers with greater exposure and broaden their familiarity with diverse MBS procedures. Early involvement in both the clinical and academic aspects of MBS may build a strong foundation in disease understanding, surgical techniques, and multidisciplinary long-term care, ultimately shaping future surgical leaders in this field.

This study has several limitations. The survey was based on a non-validated questionnaire, and the number of respondents may not accurately represent the global MBS training experience. The open dissemination method precluded calculation of a response rate and may have introduced selection bias, as residents interested in MBS may have been more likely to participate. Additionally, self-reported data were not corroborated with objective case logs or institutional records, and nonresponse bias is possible. Despite these limitations, the relatively large number of respondents allowed identification of clear regional differences. Future research using validated instruments and targeted recruitment of residents across all global regions, including North America and Africa, could yield more comprehensive insights.

## Conclusions

Significant disparities currently exist across different geographic areas, both nationally and internationally, regarding the availability of MBS services and the resulting level of resident training and exposure. To address these gaps and foster greater uniformity in MBS education, a more formalized, multi-pronged approach is necessary. We propose that the global surgical community pursue collaborative strategies that include the formal integration of MBS-specific modules into general surgery curricula. Furthermore, the development of structured global exchange programs would provide residents with invaluable clinical training in diverse geographic settings, exposing them to varying MBS practices and volumes. By implementing such targeted and collaborative initiatives, we can ensure that all general surgery residents receive comprehensive and high-quality MBS training, which is essential for improving patient care and long-term outcomes worldwide.

## Appendices

Appendix A

Questionnaire for General Surgery residents on Metabolic and Bariatric Surgery (MBS):

1)      What is your gender?

A.      Male

B.      Female

C.      Other - specify

2)      What is your age?

Answer____________

3)      What is your nationality?

Answer____________

4)      Which country do you live in?

Answer____________

5)      How many years is your General Surgery residency program?

a.       4

b.       5

c.       6

d.       7

e.       Other - Specify

6)      What year of residency are you?

Answer__________

7)      Till now, which field of general surgery interests you the most? Multiple answers

a.       Metabolic and bariatric surgery (MBS)

b.       Upper GI surgery

c.       Hepato-pancreatico-billiary surgery

d.       Surgical oncology

e.       Colorectal surgery

f.        Abdominal wall

g.       Breast surgery

h.       Endocrine surgery

8)      Have you ever thought of training in MBS after completing the residency program?

a.       Yes

b.       No

9)      Is there an MBS unit in your hospital?

a.       Yes

b.       No

10)   How many MBS procedures are performed in your center annually?

a.       <50

b.       51-100

c.       101-200

d.       201-500

e.       501-1000

f.        >1000

11)   Have you participated in MBS procedures in your hospital?

a.       Yes

b.       No

12)   If not, does your residency program offer you a clerkship elsewhere to participate in MBS procedures?

a.       Yes

b.       No

13)   Do you think participation in MBS procedures should be mandatory in a general surgery residency program?

a.       Yes

b.       No

14)   If not, why?

Answer______________

15)   How many MBS procedures have you participated till now?

a.       <10

b.       11-30

c.       31-50

d.       51-100

e.       >100

16)   If yes, what was your role in these cases?

a.       Primary surgeon

b.       Assistant

c.       Other - specify

17)   In relation to the total surgeries you participated in, how many were MBS?

a.       0%

b.       1-10%

c.       11-30%

d.       31-50%

e.       >50%

18)   Did you perform a surgical training related to MBS?

a.       Yes

b.       No

19)   If yes, what surgical training did you perform?

Answer____________________

20)   Do you think that the surgical training improved your skills?

A.      Yes

B.      No

21)   Do you have a surgical mentor?

A.      Yes

B.      No

22)   If yes, is your mentor an MBS surgeon?

a.       Yes

b.       No

23)   Do you think that the surgical mentor helped you improve your skills?

A.      Yes

B.      No

24)   Which MBS procedure did you participate in as a primary surgeon? Multiple answers

a.       Sleeve Gastrectomy

b.       Roux-en-Y Gastric Bypass

c.       One anastomosis gastric bypass

d.       Single anastomosis duodeno-ileal bypass

e.       Duodenal switch

f.        Biliopancreatic diversion

g.       Gastric band

h.       Other

i.         None

25)   Which MBS procedure did you participate in as an assisting surgeon? Multiple answers

a.       Sleeve Gastrectomy

b. Roux-en-Y Gastric Bypass

c.       One anastomosis gastric bypass

d.       Single anastomosis duodeno-ileal bypass

e.       Duodenal switch

f.        Biliopancreatic diversion

g.       Gastric band

h.       Other

i.         None

26)   In case you participated as a primary surgeon in a Sleeve Gastrectomy, which part of the procedure did you perform? Multiple answers

a.       Trocars insertion

b.       Division of omentum from the greater curvature

c.       Transection of the stomach

d.       Specimen removal

e.       Closure of wounds

27)   In case you participated as a primary surgeon in Roux-en-Y gastric bypass, which part of the steps did you perform? Multiple answers

a.       Trocars insertion

b.       Lesser omentum dissection

c.       Pouch creation

d.       Bowel measurement

e.       Anastomosis - staplers

f.        Anastomosis - suturing

g.       Closure of mesenteric defects

h.       Closure of wounds

28)   In case you participated as a primary surgeon in one anastomosis gastric bypass, which part of the steps did you perform? Multiple answers

a.       Trocars insertion

b.       Lesser omentum dissection

c.       Pouch creation

d.       Bowel measurement

e.       Anastomosis - staplers

f.        Anastomosis - suturing

g.       Closure of mesenteric defects

h.       Closure of wounds

29)   Do you see patients together with an attending surgeon in ambulatory clinics prior to surgery?

a.       Yes

b.       No

30)   Do you see patients in ambulatory clinics after surgery?

a.       Yes

b.       No

31)   Do you have a scientific mentor?

a.       Yes

b.       No

32)   If yes, is your mentor an MBS surgeon?

c.       Yes

d.       No

33)   How many scientific publications do you have?

a.       <5

b.       6-10

c.       11-20

d.       >20

34)   How many of your publications are related to MBS?

a.       0%

b.       1-10%

c.       11-30%

d.       31-50%

e.       >50%

35)   Did you get the opportunity to present at national scientific meetings?

a.       Yes

b.       No

36)   If yes, have you presented any work related to MBS?

a.       Yes

b.       No

37)   Did you get the opportunity to present at international scientific meetings?

a. Yes

b. No

38)   If yes, have you presented any work related to MBS?

a. Yes

b. No

Appendix B

**Table 5:** Participation in Sleeve Gastrectomy; Steps Performed as a Resident

Step	Number (%)
Trocar Insertion	101 (51.0%)
Division of Omentum from Greater Curvature of Stomach	80 (40.4%)
Transection of Stomach	69 (34.8%)
Specimen Removal	72 (36.4%)
Wound Closure	104 (52.5%)

Appendix C 

**Table 6:** Participation in Roux-en-Y Gastric Bypass; Steps Performed as a Resident

Step	Number (%)
Trocar Insertion	84 (42.4%)
Dissection of Lesser Omentum	45 (22.7%)
Pouch Creation	37 (18.7%)
Bowel Measurement	41 (20.7%)
Stapling of Anastomosis	39 (19.7%)
Suturing of Anastomosis	32 (16.2%)
Mesenteric Defects Closure	28 (14.1%)
Wound Closure	77 (38.9%)

Appendix D 

**Table 7:** Participation in One Anastomosis Gastric Bypass; Steps Performed as a Resident

Step	Number (%)
Trocar Insertion	72 (36.4%)
Dissection of Lesser Omentum	41 (20.7%)
Pouch Creation	27 (13.6%)
Bowel Measurement	29 (14.6%)
Stapling of Anastomosis	33 (16.7%)
Suturing of Anastomosis	30 (15.2%)
Mesenteric Defects Closure	23 (11.6%)
Wound Closure	61 (30.8%)
